# Supplementary figures and images for: Altered Electroencephalographic Resting-State Large-Scale Brain Network Dynamics in Euthymic Bipolar Disorder Patients
Source: Front Psychiatry. 2019 Nov 15;10:826. doi: 10.3389/fpsyt.2019.00826 (PMC6873781; doi:10.3389/fpsyt.2019.00826)

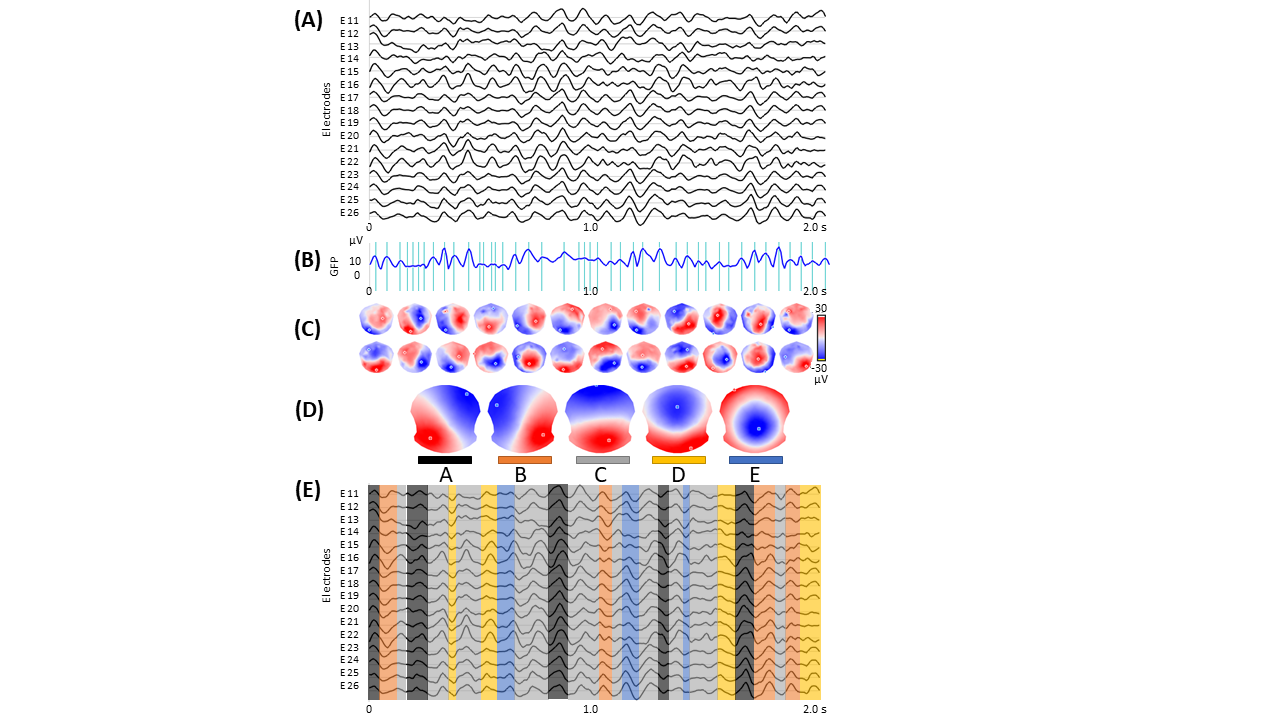

Supplement: Supplementary Figure 1 — Microstate analysis: (A) resting-state EEG from subsample of 16 out of 204 electrodes; (B) global field power (GFP) curve with the GFP peaks (vertical lines) in the same EEG period as shown in (A); (C) potential maps at successive GFP peaks, indicated in (B), from the first 1 s period of the recording; (D) set of five cluster maps best explaining the data as revealed by k-means clustering of the maps at the GFP peaks; (E) the original EEG recording shown in (A) with superimposed color-coded microstate segments. Note that each time point of the EEG recording was labelled with the cluster map, shown in (D), with which the instant map correlated best. The duration of segments, occurrence, and coverage for all microstates were computed on thus labeled EEG recording. [file Image_1.tif]
